# Do healthcare services behave as complex systems? Analysis of patterns of attendance and implications for service delivery

**DOI:** 10.1186/s12916-018-1132-5

**Published:** 2018-09-07

**Authors:** Christopher Burton, Alison Elliott, Amanda Cochran, Tom Love

**Affiliations:** 1 Academic Unit of Primary Medical Care, University of Sheffield, Samuel Fox House, Northern General Hospital, Sheffield, S5 7AU UK; 2 University of Aberdeen, Aberdeen, UK; 3 University of Otago, Wellington, New Zealand; 4 Abertay University, Dundee, UK

**Keywords:** Health services research, Emergency department, Primary care, Complex systems, Complexity, Frequent attendance

## Abstract

**Background:**

The science of complex systems has been proposed as a way of understanding health services and the demand for them, but there is little quantitative evidence to support this. We analysed patterns of healthcare use in different urgent care settings to see if they showed two characteristic statistical features of complex systems: heavy-tailed distributions (including the inverse power law) and generative burst patterns.

**Methods:**

We conducted three linked studies. In study 1 we analysed the distribution of number of contacts per patient with an urgent care service in two settings: emergency department (ED) and primary care out-of-hours (PCOOH) services. We hypothesised that these distributions should be heavy-tailed (inverse power law or log-normal) in keeping with typical complex systems. In study 2 we analysed the distribution of bursts of contact with urgent care services by individuals: correlated bursts of activity occur in complex systems and represent a mechanism by which overall heavy-tailed distributions arise. In study 3 we replicated the approach of study 1 using data systematically identified from published sources.

**Results:**

Study 1 involved data from a PCOOH service in Scotland (725,000) adults, 1.1 million contacts) and an ED in New Zealand (60,000 adults, 98,000 contacts). The total number of contacts per individual in each dataset was statistically indistinguishable from an inverse power law (*p* > 0.05) above 4 contacts for the PCOOH data and 3 contacts for the ED data. Study 2 found the distribution of contact bursts closely followed a heavy-tailed distribution (*p* < 0.008), indicating the presence of correlated bursts. Study 3 identified data from 17 studies across 8 countries and found distributions similar to study 1 in all of them.

**Conclusions:**

Urgent healthcare use displays characteristic statistical features of large complex systems. These studies provide strong quantitative evidence that healthcare services behave as complex systems and have important implications for urgent care. Interventions to manage demand must address drivers for consultation across the whole system: focusing on only the highest users (in the tail of the distribution) will have limited impact on efficiency. Bursts of attendance — and ways to shorten them — represent promising targets for managing demand.

## Background

Managing demand for healthcare is a global problem. The science of complex systems [1, 2] has been proposed as a way of understanding health services [3, 4], but there has been little quantitative evidence to support this notion. The idea that healthcare services can be considered as complex systems is not new [4–7] and remains current [3, 8], but it has rarely been tested, particularly in ways that use large-scale data. Healthcare self-evidently possesses many of the characteristics of a complex system [1, 2, 5] in that there are many component parts (patients, clinicians, services) with many interactions (consultations) which occur in the context of prevailing social attitudes and norms (e.g. ideas about when it is appropriate to seek healthcare). Because of the interactions and the way that characteristics of the system emerge from these interactions, complex systems are different from conventional systems in several ways [1, 9]. Some of these differences are listed in Table 1. Much current health services research and innovation addresses healthcare as a conventional system rather than as a complex one, with important implications for the development and implementation of complex interventions to change health and healthcare [1, 3, 9, 10].

**Table 1:** Comparison of features between a complex system and a conventional system

	Conventional system	Complex system
Relationship of individual to system	System comprises discrete individuals, who are considered as distinct and statistically independent from each other, but who share the system environment	System comprises individuals, each interacting with others in the system; characteristics of the whole system emerge from these interactions
Context and culture	Context or culture seen as separate from the individuals and may be externally directed or imposed. Treated as a confounder or covariate in analysis	Context or culture seen as emergent properties of the system. In turn, these properties condition the interactions of individuals within the system
Predictability of response to events	Multiple independent responses to change produce a coherent average value response and an approximately normal distribution	Changes to the system are usually buffered by local interaction (so have minimal effect), but sometimes events spread through the system with unexpectedly large effects
Statistical Distributions	Normal distribution for continuous measures, Poisson distribution for events	Heavy-tailed distributions for events: typically inverse power law or log-normal

Despite the resemblance of healthcare to a complex system and the wide recognition that complex systems display characteristic statistical properties [11, 12], there have been very few studies which have sought to test this by comparing the statistical properties of healthcare use with known properties of complex systems [13–15]. However, robust methods are available for this [11] which have been widely used in many other areas of science (examples include the size distributions of avalanches, forest fires and human settlements and patterns of Internet activity) [16].

One aspect of healthcare which is well suited to being examined as a complex system is the use of urgent care [17, 18]. Urgent care (emergency department (ED) and primary care out-of-hours (PCOOH) services) represents a relatively open system in which use is driven by patients rather than controlled by the service. It also includes the particular problem of high-using, or frequently attending, patients [17]. These patients take up a disproportionate amount of resources including professional time and treatment costs and are frequently portrayed as problematic individuals for whom initiatives are developed to identify and manage individual frequent attenders [19, 20]. This action at the level of the individuals carries the implication that tackling these extreme cases will resolve the pressure on urgent care services [21]. However, frequent attenders comprise a very heterogeneous group [22], including both patients who appear to need multiple attendances because of severe or complex medical conditions and others who attend for conditions that could be managed elsewhere [23] or to an extent which is disproportionate to their medical conditions [19, 24–26]. While interventions to tackle specific problems for some frequent attenders are successful at the individual level, there is little evidence that they lead to a substantial reduction in overall demand.

In contrast to the view of frequent attendance as a problem of a few individuals, a complex system perspective could argue that (1) frequent attenders might represent the “black swans [27]” occurring in the natural heavy-tailed distribution of events [11], (2) patterns of consultation by individuals over time should show the bursts typically seen in complex systems [12] and (c) there should be plausible social mechanisms which drive the behaviour of individuals across all levels of attendance from least to most frequent. While social mechanisms have been documented in several qualitative studies of healthcare seeking [28–32], there have been no studies, to our knowledge, which have examined the statistical properties of complex systems in urgent healthcare use. The closest to this have been some reports of the overall population distribution of urgent care use which described non-normal distributions [33, 34]; however, none have carried out more detailed statistical analyses.

In this study we tested the hypothesis that patterns of attendance at urgent care services should display two typical statistical characteristics of complex systems. Specifically we hypothesised first, that the overall distribution of consultations per individual would follow a power law [2, 11] and second, that individuals’ consultations would occur in correlated bursts (sequences of consultations clustered in time), with the distribution of burst lengths also approximating to a power law [12]. The implication of these hypotheses is that if urgent care services do behave as complex systems, then interventions to influence their use need to act in a system-wide fashion rather than focus on problematic individuals.

## Methods

We conducted three linked studies to compare the statistical properties of urgent healthcare use with the typical properties of a complex system. First, we defined the total number of contacts per person and compared this to two heavy-tailed distributions, the inverse power law and the log-normal. Second, we used the same data to examine the pattern of bursts of attendance. Third, we conducted a systematic search for and analysis of reports, from other centres, of the distribution of number of contacts per person to compare these results with the findings from our primary data sources.

### Data sources

We analysed primary data from two sources: PCOOH data from a study of NHS 24, the service which provides out-of-hours primary care services throughout Scotland (population 5.6 million (M)) [35] and ED data supplied by Canterbury District Health Board in New Zealand. The data was for the ED of Christchurch Hospital, serving a population of approximately 500,000 people. Both datasets were derived from routine management data and thus included all cases handled by the respective services.

In the PCOOH service, all calls were initially managed through a nurse-based triage system with a range of options including telephone advice by the nurse, consultation with a general practitioner (GP), either at a treatment centre or in the patient’s home, and direct ambulance transfer to an ED. The data included all calls to the NHS 24 service throughout 2011. We excluded calls during office hours (08.00 to 18.00 weekdays except public holidays) because the vast majority of urgent care requests during these hours go directly to the patient’s GP practice. All data was anonymised and handled under a data-sharing agreement between the University of Aberdeen and NHS 24; as no patient-identifiable data was involved, additional research ethical permission was not required. The ED data comprised anonymised data of adult attendance for the period July 2011 to June 2013. Since no identifying patient information was involved, research ethical permission was not required under New Zealand’s Standard Operating Procedures for Health and Disability Ethics Committees.

### Definitions of attendance

For both datasets, the unit of analysis was a contact — defined as one or more attendances or phone calls with the service on a given day. While some patients had more than one attendance or call on the same day, we limited data to one contact per day, since most repeated calls on the same day related to the same episode of care (e.g. escalation of a contact from telephone advice to consultation). PCOOH contacts included episodes of care which were managed by telephone, in the urgent care department or at home, and by any healthcare professionals. ED contacts included emergency attendances by any route.

### Heavy-tailed statistical distributions and correlated bursts of activity

Heavy tails are a feature of a number of statistical distributions, including the inverse power law and the log-normal, which have been repeatedly observed in large, naturally occurring systems [11]. The term refers to the long, or thick, “tail” of the distribution, in contrast to the shorter tails of distributions such as the normal, where data points cluster close to the mean (and almost always within a few standard deviations of it). One of the first described heavy-tailed distributions was the Pareto principle whereby 80% of the wealth is held by 20% of the population (and of that wealth, 80% is held by only 20% of the 20%, and so on) [16]. This observation is mathematically related to the inverse power law. The result of such a distribution is that the range of wealth from poorest to richest is much greater than would occur if individuals were normally distributed around a mean. While plots of the normal distribution produce the familiar bell curve, conventional plots of heavy-tailed distributions are difficult to interpret because of the much larger range of values. A simple solution to this is to plot cumulative distributions with logarithmic axes. Using this format an inverse power law will describe a straight line (from top left to bottom right). Heavy-tailed distributions are typically seen in theoretical complex systems and have been held to be a typical feature, or fingerprint, of complex systems [11].

While there are many possible explanations for the occurrence of heavy-tailed distributions [36], and their presence alone is not proof of the presence of a complex system, the more recent description of correlated bursts of activity within a complex system leading to heavy-tailed overall distributions [12] means that identifying both in the same data strengthens the evidence for the presence of a complex system. Correlated bursts of activity represent clusters of activity in time, with the distribution of burst lengths also following a heavy-tailed distribution [12]. This distribution of bursts means that long bursts arise more often than would occur by chance, and furthermore that bursts undergo a kindling process, whereby the longer a burst has gone on, the more likely it is to continue.

### Study 1: analysis of total contacts per patient

We conducted this analysis using the total number of contacts per patient in 1 year of data from each dataset. We plotted the complementary cumulative distribution function (CDF), defined as the proportion of patients whose total number of contacts was equal to or greater than each number of contacts between 1 and the largest recorded. This is preferable to plotting the probability distribution function, as the CDF is more robust against fluctuations in the tail of the distribution due to finite sample sizes [11]. Plots of the CDF were made with logarithmic axes, on which a power law distribution displays a straight line. The slope of this line is equal to one minus the scaling parameter of the power law distribution (for example, a scaling parameter of 3 produces a slope of − 2). We fitted inverse power law and log-normal functions to each dataset using maximum likelihood fitting with the poweRlaw package in R 3.3.2. We examined the closeness of fit of the data to a power law distribution, using the Kolmogorov-Smirnoff (KS) test. Where data was deemed different from a power law distribution (*p* value < 0.05) we examined whether the data was closer in fit to a power law or log-normal distribution using the Vuong test for non-nested distributions [37] at a significance level of 0.01. As naturally occurring distributions often include a power law only above a certain threshold [11], we repeated this analysis with a range of minimum values for the number of contacts per patient.

#### Subgroup and sensitivity analysis

We conducted a subgroup analysis of the PCOOH data with data split by sex and by age using the median value. For each subgroup we repeated the plotting and model-fitting procedure described above, with the addition of 95% confidence intervals (CIs) derived from a non-parametric bootstrapping procedure (1000 iterations). We did not conduct a subgroup analysis of the ED data because of the smaller number of patients.

We conducted two post hoc sensitivity analyses to test for artefacts arising from the fact that each dataset contained a calendar year but that patients might have their first consultation at any point during the year and thus have different durations of follow-up. In the first sensitivity analysis we limited data to patients consulting within the first half of the year and censored each patient’s data 6 months after their first contact. In the second sensitivity analysis we considered that some patients consulting in the first few weeks of the year would be doing so within a burst of contacts. We thus split the PCOOH data into patients whose first contact was before the 15th day of the time period and those whose first contact was on or after the 15th day. In each of the sensitivity analyses we used the same method for plotting and analysing distributions as described for the whole dataset.

### Study 2: analysis of bursts of contacts

We examined bursts of contacts using the method developed by Karsai [12]. We defined a burst as a sequence of contacts in which the interval between each pair of consecutive contacts is less than a specified time window *∆t.* We used a range of values for *∆t* of 4 and 7 days for the PCOOH data and 4, 7 and 10 days for the ED data. For each burst we counted the number of contacts within the burst. We pooled the burst patterns across all individuals and limited the analysis to patients with between 4 and 52 contacts in order to exclude data with too few contacts to display bursts, or so many contacts that bursts were inevitable because the PCOOH data comprised only 1 year. We conducted the burst analysis on the PCOOH dataset (1 year) and on the whole ED dataset (2 years).

For each dataset and value of *∆t* we plotted the CDF. As for the distribution of total contacts, we plotted the CDFs using logarithmic axes, so that an inverse power law would display as a straight line. In order to assess whether the burst patterns could have arisen by chance, we conducted a bootstrapping procedure in which we compared the actual data with a set of surrogate datasets in which the temporal structure of bursts was broken by randomly allocating the number of contacts for each patient to dates within the time between their first contact and the end of the study period. These surrogate datasets were thus identical to the original dataset except that the bursts they contained arose at random. We conducted this bootstrapping procedure with 250 iterations, meaning that the probability of any observed distribution lying wholly above the bootstrapped range by chance was less than 0.008.

### Study 3: review of data from published reports

We systematically identified papers reporting numbers of patients attending EDs, using a structured search of the MEDLINE and Embase databases (Table 2 lists the search terms). Titles, abstracts and full manuscripts were screened by two authors independently to identify usable data. We followed references from, and citations of, eligible papers to identify additional studies.

**Table 2:** Search terms

Emergency department	
1) Emergency Service, Hospital/	
2) ((emergency or casualty) adj1 department).mp.	
3) (accident adj2 emergency).mp.	
4) 1 or 2 or 3	
5) (frequen$ adj2 attend$).mp.	
6) “high use$”.mp.	
7) (hig$ adj (utiliz$ or utilis$)).mp.	
8) “frequent flier”.mp.	
9) (frequen$ adj3 use$).mp.	
10) 5 or 6 or 7 or 8 or 9	
11) 4 and 10	
Primary care out-of-hours service	
1) General Practice/	
2) Primary Health Care/	
3) “General Practi$”.mp.	
4) “GP”.mp.	
5) “primary care”.mp.	
6) 1 or 2 or 3 or 4 or 5	
7) (out adj2 hours).mp.	
8) “out-of-hours”.mp.	
9) “unscheduled”.mp.	
10) 7 or 8 or 9	
11) (frequen$ adj2 attend$).mp.	
12) “high use$”.mp.	
13) (hig$ adj (utiliz$ or utilis$)).mp.	
14) “frequent flier”.mp.	
15) (frequen$ adj3 use$).mp.	
16) 11 or 12 or 13 or 14 or 15	
17) 6 and 10 and 16	

#### Inclusion and exclusion criteria

We included studies which reported urgent care attendance data either in EDs or PCOOH services. We required reports to include all of the following: setting (time and place), an unselected population (e.g. “all attenders” or “all adults”, but not “adults with asthma”) and a continuous or categorical (binned) distribution of individual patient attendances over 1 year which included all attenders. We excluded studies which reported less than four categories or where the lower threshold of the highest category was less than 10 episodes of care, in order to ensure a spread of data points and include at least one order of magnitude for the number of episodes of care. Where a study reported more than 1 year or more than one site for care separately, we used the most recent year or the largest site. Where studies reported several sites together, we did not attempt to separate them. Studies varied in the categories they used to report attendance (individual numbers of attendances, ranges of attendances or a mixture of the two). In most cases we kept data in the original format; where studies reported many categories, each with small numbers (< 10) of individuals, we aggregated them into category ranges containing 10 or more individuals. We did not restrict studies on the basis of healthcare system or level of economic development.

#### Quality assessment of included studies

All studies were observational studies describing similar retrospective data collection of a complete sample. Provided studies met our stringent inclusion and exclusion criteria we did not apply further quality assessments, as the topics for evaluation in common tools (e.g. completeness of sample, sources of bias, etc.) are designed for studies which make inferences based on samples from populations, whereas the studies we included reported on counts of attendance for entire services.

#### Distributions of attendance per patient in review data

For each study we plotted the complementary CDF: the proportion of patients whose total number of attendances was equal to or greater than the lower boundary of each category. Plots used logarithmic axes to facilitate the display of heavy-tailed data. We plotted data for ED and PCOOH studies separately. In addition, we selected a subset of studies which contained at least 8 data bins, with the highest data bin threshold set ≥ 20. As most studies provided heavily aggregated data with wide categories, we did not attempt to fit distributions to this data.

## Results

### Analysis of total contacts per patient

Primary data was available from 724,921 PCOOH patients (1,085,796 contacts) and 60,106 ED patients (98,228 contacts). The age and sex characteristics and number of contacts per patient are listed in Table 3.

**Table 3:** Characteristics of patients in PCOOH and ED datasets

	PCOOH patients (*N* = 724,921)	%	ED patients (*N* = 60,106)	%
Age				
< =5	119,611	16.5	7482	14.5
6–17	78,757	10.9	6414	12.4
18–45	263,234	36.3	17,905	34.6
46–70	154,826	21.4	12,361	23.9
71+	108,486	15.0	7524	14.6
Not recorded	7	–	8420	–
Sex				
Female	420,663	58.0	–	–
Male	304,258	42.0	–	–
Number of contacts				
1	532,807	73.5	40,011	66.6
2–5	181,906	25.1	18,965	31.6
6–10	8105	1.1	951	1.6
11–50	2002	0.3	177	0.3
51–100	79	0.01	2	0.003
101+	22	0.003	0	–

**Fig. 1:**
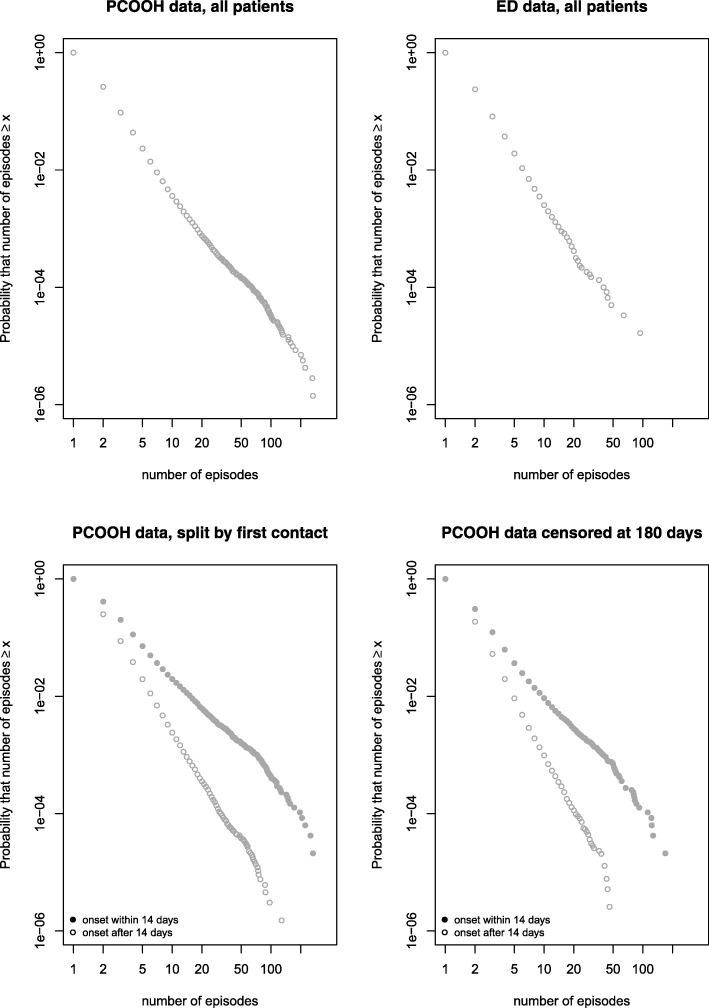
Plots of the distribution of contacts per patient for (**a**) Primary Care Out of Hours Service (PCOOH); (**b**) Emergency Department (ED); (**c**) PCOOH split by date of first contact to separate those with at least 14 days of no contact before their first contact (**d**) PCOOH censoring data so all patients had 26 weeks data after their first contact

Plots of total number of contacts per individual are shown in Fig. 1a (PCOOH data) and 1b(ED data). Both plots show a heavy-tailed distribution, which approximates to an inverse power law (straight line) for the whole distribution in the ED data and from approximately 5 contacts to 30 contacts in the PCOOH data. Above 30 contacts in the PCOOH data (Fig. 1a) the tail of the distribution can be seen to deviate from the power law; there were more patients than expected with very high numbers of contacts: 225 patients (0.03%) had more than 30 contacts. This represents approximately twice as many as would have been expected if the data followed a power law distribution. This pattern is suggestive of more than one overlapping distribution. Figure 1c shows the result of the sensitivity analysis in which the PCOOH data was split into patients whose first contact occurred within the first 14 days of the year and those whose first contact came later. The rationale was that patients consulting in the first 14 days might be within a burst of consultations at the start of the data collection and thus might be more likely to have repeat consultations than those starting their first burst after at least 14 days of no contact. The two resulting distributions in Fig. 1c both showed close approximation to a power law. Finally, Fig. 1d shows the analysis repeated with censoring of data at 6 months after the first consultation, indicating that this had no adverse influence on the observed distribution’s approximation to a power law.

#### Statistical model fitting

Table 4 lists the statistical parameters from the fitting of inverse power law and log-normal distributions to the data. Values for PCOOH (first contact after the first 14 days) and ED data were broadly similar, and for patients with 5 or more contacts both distributions showed good fit to a power law (KS test *p* value > 0.05) with similar exponents of 3.8 and 3.7.

**Table 4:**
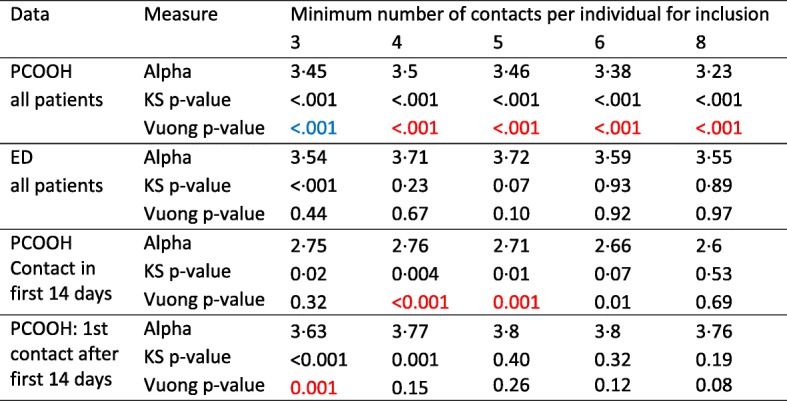
Power Law scaling parameter and tests of fit for selected distributions by minimum value of contacts included in analysis

*PCOOH* primary care out-ofhours (service), *ED* emergency department

*Alpha* represents the scaling parameter of the power law probability distribution *p*(*x*) ∝ *x*^−*α*^

*KS* Kolmogorov-Smirnoff test for fit of data to power law, reported as *p* value (value > 0·05 indicates no difference between data and power law)

*Vuong* Better fitting distribution to the data by Vuong test (*p* values in blue indicate that the power law was the better fitting distribution, *p* values in red that the log-normal was the better fit

The good fit of the power law (and log-normal) distributions to the whole population supports the hypothesis that urgent healthcare systems show one of the typical statistical characteristics of complex systems. Despite the occurrence of extreme frequent attenders (the maximum number of contacts was 266 and 94 in the PCOOH and ED data respectively), the proximity of these extreme points to the fitted curves shows that these events occurred with the expected frequency for their respective distributions. This suggests that frequent attenders are indeed the “black swans” which naturally occur in complex systems [27].

#### Subgroup analysis

**Fig. 2:**
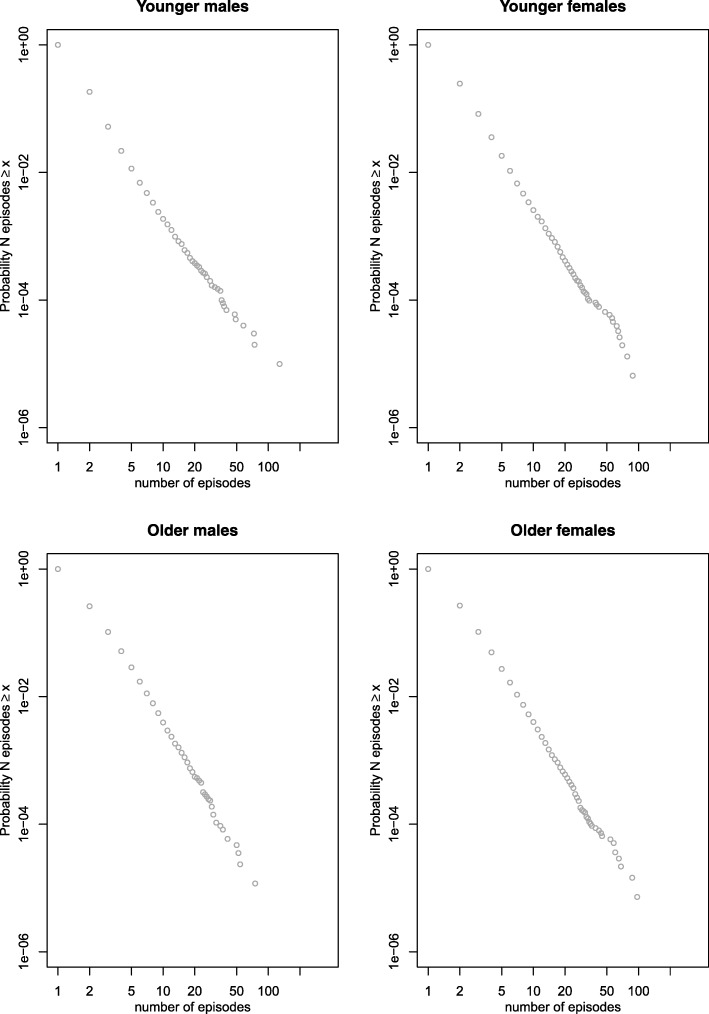
Plots of the distribution of contacts per patient for primary care out of Hours by age and sex subgroups

The subgroup analysis, by age and sex, is reported in Table 5 and Fig. 2. The figures and data indicate that the distributions were heavy-tailed in each subgroup, but that the scaling parameter was larger (a steeper gradient on the plots) in younger than older adults. There was less difference between the sexes.

**Table 5:** Power law scaling parameter (alpha) by minimum value of contacts included in analysis in subgroups of patients split by sex and by median age

Inclusion	Subgroup	Minimum number of contacts per individual for inclusion			
		Minimum 3 contacts		Minimum 5 contacts	
		Alpha	95% CI	Alpha	95% CI
	Younger male	3.35	3.28–3.41	3.05	2.94–to 3.16
All patients	Younger female	3.42	3.38–3.46	3.29	3.21–3.38
	Older male	3.13	3.09–3.17	3.31	3.22–3.39
	Older female	3.19	3.15–3.22	3.24	3.17–3.31
Patients with first contact after 14th day	Younger male	3.60	3.52–3.67	3.42	3.28–3.57
	Younger female	3.62	3.57–3.67	3.65	3.55–3.75
	Older male	3.30	3.26–3.35	3.64	3.54–3.74
	Older female	3.37	3.34–3.41	3.58	3.50–3.67

Alpha: scaling parameter of the power law probability distribution *p*(*x*) ∝ *x*^−*α*^

95% confidence intervals (CIs) derived by non-parametric bootstrapping with 1000 iterations

### Analysis of bursts of contacts

**Fig. 3:**
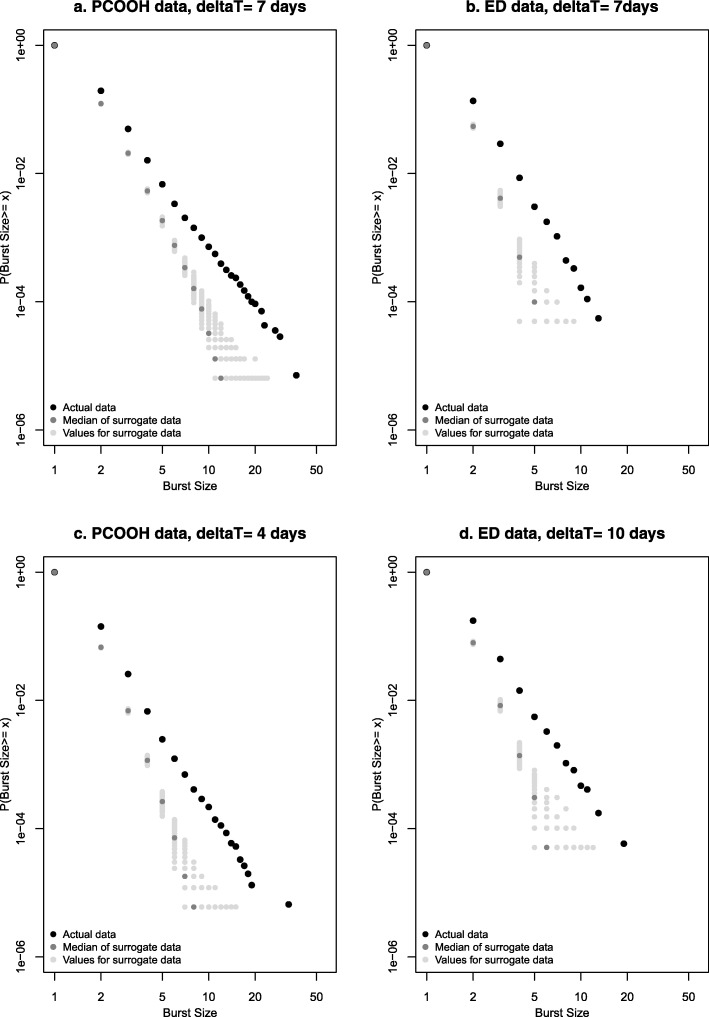
Distribution of burst lengths in original data and in bootstrapped surrogate data (250 iterations): (**a**) Primary Care Out of Hours (PCOOH) data with time window Δ*t* = 7 days; (**b**) Emergency Department (ED) data with Δ*t* = 7 days; (**c**) PCOOH data with Δ*t* = 4 days; and (**d**) ED data with Δ*t* = 10 days

Plots of the distribution of burst length are shown in Fig. 3a and b for both the PCOOH data and ED data. Both plots use a 7-day window for inclusion of contacts within bursts. Both distributions are clearly heavy tailed, approximating to a straight line indicative of an inverse power law. None of the 250 surrogate datasets, in which the temporal structure of bursts was disrupted, showed this distribution, suggesting that it was unlikely to have arisen in the data by chance. Similar patterns were seen from the PCOOH data with a 4-day window (Fig. 3c) and from the ED data with 4- and 10-day windows (Fig. 3d). This similarity across different time windows makes it unlikely that the observed results were due to an artefact of the measurement parameters and more likely that these new findings represent real phenomena present in the data.

### Systematic analysis of data from published reports

#### Included studies

**Fig. 4:**
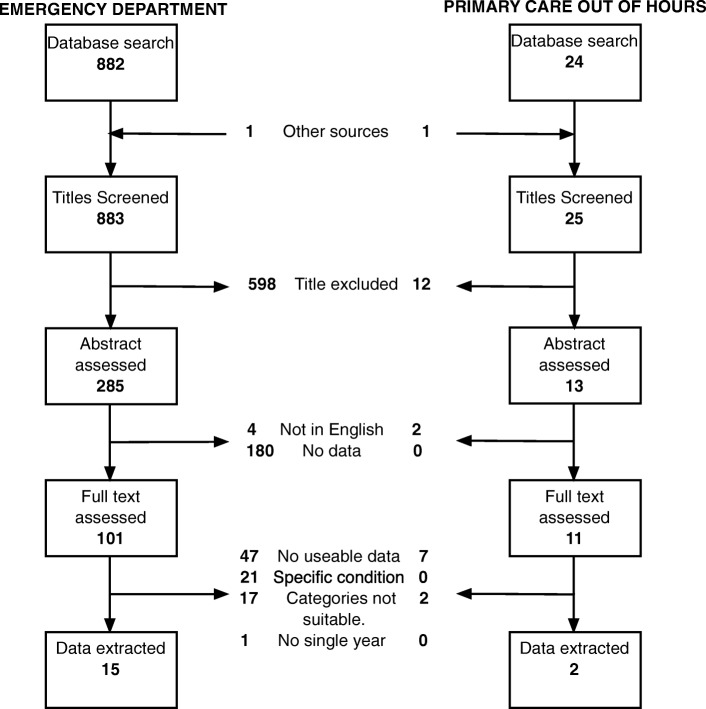
Flowchart for identification of studies for inclusion in secondary data analysis

We identified 883 titles from the search of ED attendance, from which 15 studies contained data suitable for analysis. We also identified 25 titles relating to out-of-hours primary care, resulting in two studies with data suitable for analysis. Flowcharts of the selection process are shown in Fig. 4. Characteristics of the included studies are summarised in Table 6. Briefly, studies dated from between 1999 and 2015. Eight were from single EDs (range of sample size 22,492–95,170) [19, 33, 38–43]; six from multiple departments in the same city (range 13,959–212,959) [34, 44–48]*;* and one from a network of departments (*N* = 930,712) [49]. Eight ED studies were from the USA [39, 40, 42, 43, 46–49], two from the UK [19, 33] and one each from Canada [45], Australia [44], Singapore [41], the Netherlands [34] and Ireland [38]. One PCOOH study was from the Netherlands (44,953 patients) [50] and one from Italy (17,657) [51].

**Table 6:** Characteristics of studies included in secondary data analysis

Author	Year	Location	Number of departments	Study Population	Total patients	Attendance categories	Highest category
Emergency department							
Van der Linden [34]	2014	Netherlands	2	All	51,272	14	34
Billings [48]	2013	USA	Multiple	Medicaid, ages 18–62	212,259	7	15+
Capp [47]	2013	USA	1 + satellites	Medicaid, all ages	13,959	4	18+
Doran [49]	2013	USA	Network	Veterans, Veterans Health Administration insurance	930,712	5	26+
Liu [43]	2013	USA	1	All	65,201	4	19+
Martin [42]	2013	USA	1	All	95,170	5	20+
Minassian	2013	USA	2	All	39,249	20	41+
Doupe [45]	2012	Canada	6	Age 18+	105,688	12	18+
Paul [41]	2010	Singapore	1	All — if has not attended the ED in past 12 months	82,172	6	10+
Moore [19]	2009	UK	1	All	82,812	6	10+
Locker [33]	2007	UK	1	All	75,141	16	16+
Jelinek [44]	2008	Australia	9	Age 15+	186,069	6	40+
Ruger [40]	2004	USA	1	All	50,850	5	21+
Riggs [39]	2003	USA	1	All	22,492	17	35
Murphy [38]	1999	Ireland	1	All	34,908	13	21+
Primary care out of hours							
Buja [51]	2015	Italy	1	All	17,657	11	11+
Den Boer-Wolters [50]	2010	Netherlands	1	All	44,953	10	10+

#### Distribution of contacts per patient from included studies

**Fig. 5:**
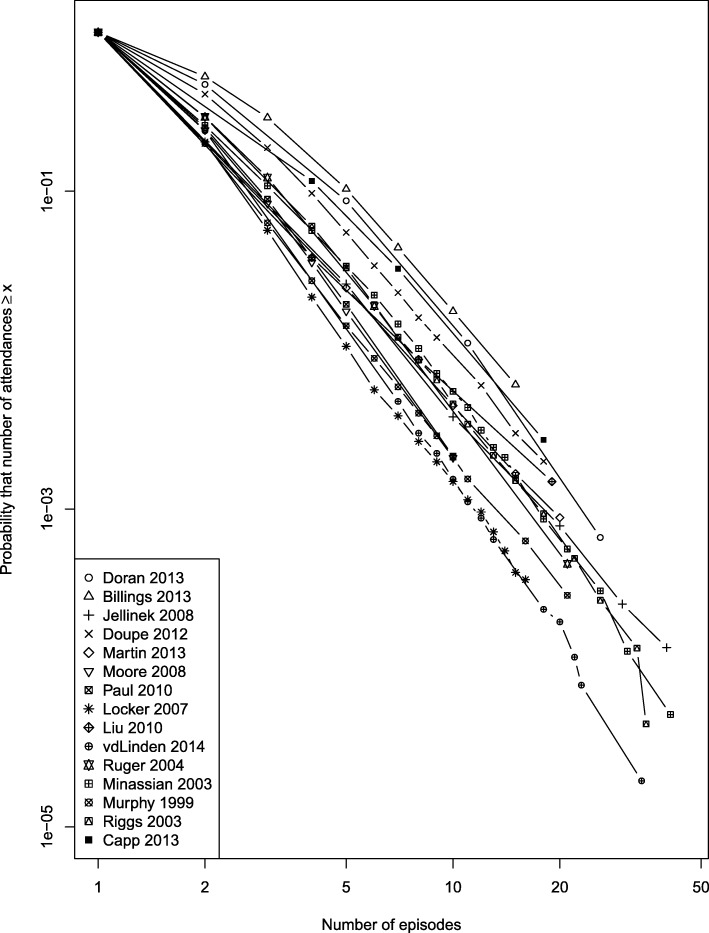
Cumulative distribution function of urgent care episodes per patient in individual study reports: all emergency department studies

**Fig. 6:**
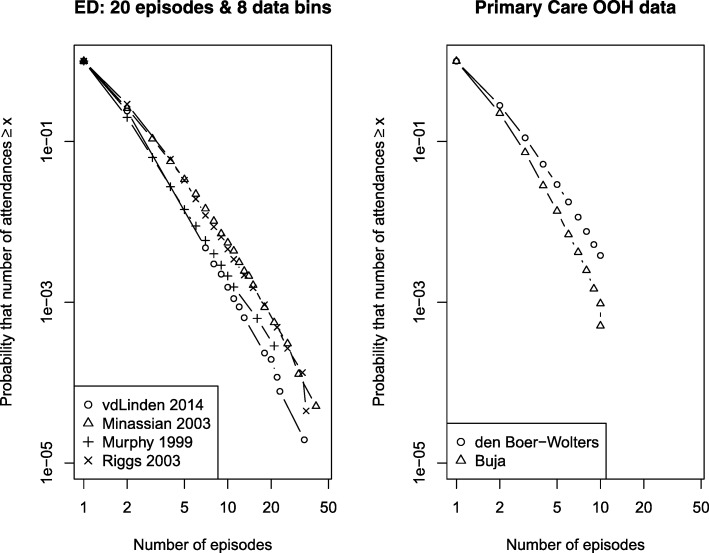
Cumulative distribution function of urgent care episodes per patient in individual study reports: **a** emergency department studies with more stringent eligibility criteria, **b** primary care out-of-hours studies. *ED* emergency department, *OOH* out of hours

Figure 5 shows data from the 15 ED studies. In each plot, the distribution was typical of a heavy-tailed distribution, and for all but one study (which included pooled patient data from multiple sites [49]) followed an approximately straight line above 3 episodes, suggesting a power law. Figure 6a shows a subset of four studies which met more stringent criteria of reporting at least 8 data bins and with a threshold for the highest bin of at least 20 attendances. These studies all show distributions similar to those in our primary data. Finally, Fig. 6b shows the two primary care studies.

The similarity of the distributions across location, healthcare type (free at the point of delivery, paid/insured) and time (almost 20 years) suggests that the patterns we observed are consistently present and represent a characteristic property of urgent care systems. While we did not fit statistical models to the data (because the effect of binning meant that the data was too sparse), the data in Fig. 6 can be compared with the more detailed data in Fig. 1. Simple visual comparison of the plots indicates that for the ED data in Fig. 1b, 1 in 10,000 patients (*y* = 10^− 4^) had 30 or more contacts, whereas in Fig. 6a, a similar proportion had between 20 + and 30+ more contacts. This suggests that our detailed dataset was broadly comparable to the other published but less detailed series.

## Discussion

This data provides original and robust evidence that patients using urgent care do so in patterns typical of individuals within a complex system. This evidence is present in both the distribution of bursts of contacts by individuals and in the overall distribution of contacts per individual. Finding both features together is important, as bursts of contact are a plausible generative mechanism for the overall distribution [12]. Frequent attenders occurred with a frequency which was in keeping with the hypothesised statistical distributions.

### Strengths and limitations

This study used large, recent and complete datasets from two different urgent care settings in different healthcare systems. The analysis used established techniques for burst estimation [12] and model fitting [11]. We also adjusted for different lengths of follow-up by censoring data and found it had no influence on the findings. Examining the combination of both burst analysis and overall distribution analysis is important, as bursts have been identified as a generative mechanism for power laws in other systems. Furthermore, bursts have been identified in other healthcare research, such as the tendency of exacerbations of chronic obstructive pulmonary disease to cluster in time [52].

While the ED data showed a close fit to a power law across the whole range of contact numbers, there was some evidence that the PCOOH data contained more very frequent attenders (above 30 contacts) than expected from the best fitting model. This may indicate some excessive or inappropriate use, but the absolute number of patients was small. When we restricted the analysis to patients who did not use the service in the first 2 weeks of the year (and so who were not currently in a burst of consultations), the observed data showed a closer fit to an inverse power law.

The inclusion of the systematic identification of secondary data adds strength to our findings of overall distributions, as heavy-tailed distributions of use, similar to those seen in our primary data, were observed across very different healthcare settings, with generally similar parameters for the proportion of frequent consultation. We were not able to conduct statistical analysis on these secondary sources of data, as they did not have sufficient detail.

### Relationship to other research

While complex systems have been hypothesised as a way of describing healthcare services [3–5, 7, 13], this is the first large-scale empirical examination of whether urgent healthcare displays the typical statistical properties of a complex system. No previous studies have reported the population distribution of urgent care attendance in detail; however, non-normal distributions of use have been previously noted but not analysed in the ways we have used in this study [33, 34].

To be plausible, our finding of the typical statistical properties of a complex system must be compatible with real-life mechanisms, which in modelling of social systems can be considered as rules [5, 53]. Qualitative studies have already suggested candidate rules: patients simultaneously seek to balance being a prudent user of services [29] with being “better safe than sorry” [30], and this balance is influenced by societal processes and norms [31, 32]. In turn, these rules may be mediated through processes such as candidacy (seeing oneself as an appropriate user of services) and recursivity (a tendency to repeat patterns of help-seeking which have been successful) [28]. Together, these processes — which are socially mediated — can be seen as comprising system-wide mechanisms which drive, and restrain, urgent care use by individuals.

Frequent attendance is commonly regarded as abnormal and taken to be a sign of an inefficient system, however many frequent attenders appear to use healthcare appropriately [17], suggesting the system may in fact be operating efficiently. Recent work in information theory suggests that power law distributions may represent an optimal configuration for a system to meet very variable demands [54]: in the case of urgent care, systems must deal with many patients with minor problems while retaining the capacity to handle a few with intensive ones. Heavy-tailed distributions of attendance may be a feature of well-optimised urgent care rather than a sign that something is wrong.

### Implications for policy, practice and research

Our findings of striking similarity between data from urgent care use and statistical features of typical complex systems support the argument that services need to engage more with a complex systems approach [3]. This means there should be a greater focus on contextual matters across the whole system and a recognition that the mechanisms driving processes such as demand both arise from, and influence, many individual interactions. In turn, this means there is a need for interventions to influence these mechanisms, which are social, both through information channels and media, and through creating and sharing positive patient experiences. A second general consequence of considering healthcare systems as complex is that interventions to change services must recognise that complex systems respond unpredictably to interventions to change them [3, 5, 15], and that what works in one setting will not necessarily work in another. This dependence on context is still under-acknowledged in the development of “complex interventions” [3], which should be viewed as “interventions in complex systems” [55].

In practice, the implication of our findings for frontline care is that there must be a partial shift in thinking from individual frequently attending patients to the workings of the whole system. While each frequently attending individual is unique, the consistent and mathematically predictable frequency with which they occur is highly suggestive of overall system effects. In theoretical models of complex systems, this dependence on system effects means that even if extreme outliers (such as individual frequent attenders) are removed (representing action on individuals), new ones will arise to fill their place [56]. This phenomenon can be seen in waiting lists — whereby initiatives to shorten them (by bringing forward treatment of individuals) generally lead to them rapidly re-growing through system effects [57, 58]. Services thus need to provide care which is simultaneously person-centred and system-aware.

For research, our identification of bursts represents a potential target for interventions to identify and respond to individuals with high need. Interventions should be developed to prevent, or shorten, bursts. These interventions must be safe, while addressing the mechanisms by which patients rationalise decisions to consult, such as candidacy and recursivity [28]. This may involve forms of explanation or sign-posting which make patients more likely to use alternative management the next time a situation occurs rather than more likely to re-attend the urgent care service, as currently happens. A focus on recognising bursts at an early stage may also permit identification of individuals at high risk of frequent attendance. In our ED data, among people who attended at least four times in a year, a burst of 3 consultations each separated by no more than 7 days represented only 1% of bursts. In the PCOOH setting, bursts of 4 consultations each separated by no more than 7 days accounted for 1% of bursts. These may represent useful “early warnings” of emerging problems, and these and other potential signals of ongoing high use should be tested in further analyses.

## Conclusions

We have demonstrated new and widespread evidence of typical complex system behaviour in urgent care use, particularly in the links between bursts of attendance and overall demand. Interventions to address demand must reflect this, by addressing systemic processes across all levels of use and by safely reducing re-attendance to shorten bursts of contacts which act as a major driver of heavy use.

## Abbreviations

CDF: Cumulative distribution function
CI: Confidence interval
ED: Emergency Department
KS: Kolmogorov Smirnoff
NHS: (UK) National Health Service
NHS24: NHS Scotland Primary Care Out of Hours Service
PCOOH: Primary Care Out of Hours Service
